# Structural diversity and chemical space analysis of a PROTAC database using unsupervised machine learning

**DOI:** 10.1038/s41598-026-53215-x

**Published:** 2026-06-10

**Authors:** Ashutosh Kharwar, Alberto Marbán-González, José L. Medina-Franco, Carlos A. Velázquez-Martínez

**Affiliations:** 1 Faculty of Pharmacy and Pharmaceutical Sciences, University of Alberta, Edmonton, AB T6G 2E1 Canada; 2 Department of Chemistry, Babasaheb Bhimrao Ambedkar University, Lucknow, 226025 Uttar Pradesh India; 3 DIFACQUIM Research Group, Department of Pharmacy, Faculty of Chemistry, Universidad Nacional Autónoma de México, Mexico City, 04510 Mexico

**Keywords:** PROTACs, Chemoinformatics, Chemical space, Unsupervised learning, Drug discovery, Biochemistry, Chemistry, Computational biology and bioinformatics, Drug discovery

## Abstract

Targeted protein degradation (TPD) mediated by proteolysis-targeting chimeras (PROTACs) has emerged as a powerful therapeutic strategy, enabling the catalytic and selective elimination of disease-relevant proteins via the ubiquitin–proteasome system. Despite their ability to overcome drug resistance and address traditionally undruggable targets, the structural complexity and vast chemical diversity of PROTACs present challenges for systematic analysis and rational design. Here, we present a systematic unsupervised machine-learning framework that represents the first large-scale similarity-driven clustering and scaffold-centric analysis of the PROTAC chemical space, to comprehensively characterize the structural, functional, and physicochemical landscape of PROTAC molecules and to support data-driven lead optimization and next-generation degrader design. An initial dataset of 9,380 compounds was curated from the publicly available PROTACs Database (PROTAC-DB 3.0), followed by rigorous standardization and filtering, resulting in 6,113 unique, chemically valid compounds. The chemical space was explored using a multi-step computational pipeline involving dimensionality reduction and a comparative evaluation of diverse clustering algorithms. Among the evaluated approaches, a refined clustering strategy demonstrated superior performance in partitioning the dataset into structurally coherent groups. Structural analysis revealed a pronounced convergence around canonical PROTAC architectures, characterized by conserved E3 ligase-binding motifs, diverse target-binding frameworks, and heterogeneous linker designs. Functional group profiling and physicochemical analysis further demonstrated that these compounds predominantly occupy a specialized chemical space beyond traditional drug-like limits, marked by high molecular weight and substantial conformational flexibility. Collectively, these findings provide data-driven design guidance for PROTAC optimization by highlighting frequent scaffold architectures and preferred property ranges, thereby informing the development of next-generation degraders.

**Supplementary Information:**

The online version contains supplementary material available at 10.1038/s41598-026-53215-x.

## Introduction

The selective modulation of protein function has traditionally relied on small-molecule inhibitors that operate through occupancy-driven mechanisms. Numerous approved drugs and advanced clinical candidates reside within the biologically relevant chemical space, as systematically defined by the BIORECs framework, which is based on compounds with demonstrated biological activity. Nevertheless, this paradigm remains limited by the need for sustained target engagement, vulnerability to resistance-conferring mutations, and limited applicability to proteins lacking well-defined binding pockets^1–3^. As a result, a large fraction of the human proteome, particularly transcription factors, scaffolding proteins, and regulatory complexes, remains inaccessible to conventional pharmacological intervention. Targeted protein degradation (TPD) has emerged as a paradigm‑shifting therapeutic strategy that overcomes many of these limitations by eliminating disease‑relevant proteins rather than merely inhibiting their function^4–6^. Among TPD approaches, proteolysis-targeting chimeras (PROTACs) have gained exceptional attention due to their modular, heterobifunctional design and broad applicability across diverse protein classes^7–9^.

**Fig. 1:**
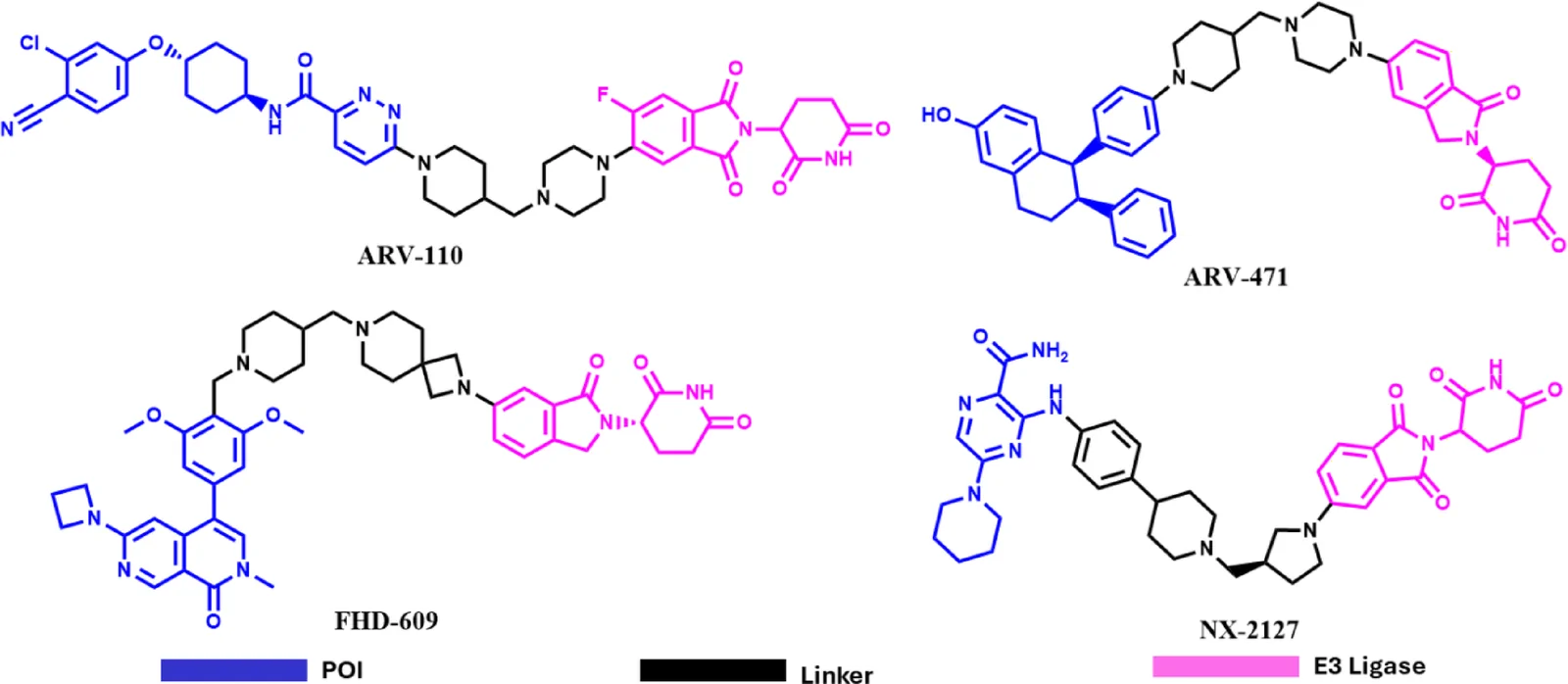
Chemical structures of PROTAC degraders in phase i and phase ii clinical trials.

PROTACs are composed of three key elements: a ligand that binds the protein of interest (POI), a ligand that recruits an E3 ubiquitin ligase, and a chemical linker that spatially connects these two moieties. By inducing proximity between the POI and an E3 ligase, PROTACs promote ubiquitination of the target protein, leading to its subsequent degradation by the ubiquitin–proteasome system (UPS)^10–12^. A defining advantage of PROTACs lies in their event-driven and catalytic mode of action. Unlike traditional inhibitors that require continuous target occupancy to maintain efficacy, PROTACs can induce multiple cycles of protein degradation from a single binding event, resulting in sustained target depletion even after compound clearance^13–15^. This catalytic behaviour enables lower effective dosing, prolonged pharmacodynamic effects, and reduced susceptibility to resistance mechanisms arising from target overexpression or mutation^16^. Importantly, PROTACs enable the degradation of proteins previously considered undruggable, including transcription factors and non-enzymatic signalling proteins. This potential is reflected in the advancement of several PROTAC degraders shown in Fig. 1, including ARV-110 and ARV-471, which target the androgen and estrogen receptors, respectively, and are currently in Phase II trials, as well as DT2216, a B-cell lymphoma extra-large -BCL-XL- degrader, and NX-2127, a -Bruton’s tyrosine kinase- BTK degrader, which have progressed into Phase I trials^17–20^.

Despite these advantages, PROTAC development faces significant chemical and pharmacological challenges. Their bifunctional architecture often results in high molecular weight, increased polarity, and pronounced conformational flexibility, placing PROTACs beyond classical drug-likeness guidelines such as Lipinski’s Rule of Five and more recent beyond-Rule-of-Five (bRo5) and chameleonicity-based frameworks^21,22^. These properties complicate the optimization of permeability, bioavailability, and metabolic stability. In addition, PROTAC activity depends not only on individual binding affinities but on the formation of a productive ternary complex between the target protein and the recruited E3 ligase. This process is highly sensitive to linker architecture, making PROTAC design intrinsically complex^23–26^.

To date, PROTAC research has predominantly focused on a limited subset of E3 ligases, most notably cereblon (CRBN) and von Hippel–Lindau (VHL), despite the existence of over 600 E3 ligases encoded in the human genome^27,28^. Expanding the diversity of ligase recruiters and systematically understanding how different ligase–target combinations shape PROTAC chemical space remain critical challenges for the field. At the same time, the rapid growth of reported PROTACs in both academic and industrial settings has generated increasingly large and chemically diverse datasets, providing an unprecedented opportunity for data-driven analysis. Computational and machine-learning approaches began to play an important role in PROTAC research. Here, computational approaches encompass structure‑based and ligand‑based methods, including molecular modelling and simulation. In contrast, machine‑learning approaches rely on data‑driven statistical models to learn patterns from existing PROTAC datasets. Together, these strategies have been applied to predict degradation efficiency, optimize physicochemical properties, and model ternary complex formation^29,30^. However, most existing studies rely on supervised learning approaches that require high-quality labelled datasets, which are scarce and highly heterogeneous in the PROTAC domain. This heterogeneity largely arises from inter-assay variability, including differences in experimental duration, enzyme and substrate concentrations, and response measurement methods, thereby introducing systematic biases that are exacerbated by the lack of standardized protocols. For example, public ChEMBL data for FOXM1 Degrader are restricted to 101 compounds (ChEMBL IDs CHEMBL5247636 and CHEMBL5052830)^31^.

In contrast, unsupervised machine-learning approaches, data-driven algorithms that identify patterns without requiring experimental measurements of degradation potency or efficacy, and enable systematic exploration of PROTAC chemical space defined by the structural and physicochemical diversity of degrader compounds. Despite their utility elsewhere, systematic unsupervised analyses of PROTAC datasets are lacking, limiting global understanding of PROTAC chemical space. This study addresses this gap by providing an unbiased structural organization of reported PROTACs to support rational exploration and future design efforts.

Addressing this gap is essential for advancing rational PROTAC design beyond empirical trial-and-error approaches. A comprehensive, unsupervised characterization of PROTAC chemical space can reveal frequent architectural motifs, underexplored scaffolds, and property regimes associated with successful degraders, thereby guiding scaffold hopping, linker engineering, and modular recombination strategies.

In this study, we address this unmet need by applying a systematic chemoinformatic workflow that leverages unsupervised machine-learning techniques to interrogate the chemical space of proteolysis-targeting chimeras at scale. Starting from a large, curated PROTAC dataset assembled from the publicly available PROTAC-DB (comprising 6,111 PROTACs, 570 target-binding warheads, 107 E3 ligands, and 2,753 linker architectures), molecular structures are rigorously standardized and encoded using circular Morgan fingerprints to capture relevant topological features. Dimensionality reduction is subsequently employed to distil the most frequent variance in the calculated fingerprints from the dataset, followed by unsupervised, similarity-based clustering using multiple complementary algorithms to organize PROTACs into structurally coherent groups objectively. Quantitative cluster evaluation based on chemical similarity and scaffold coherence enables the identification of frequent chemotypes and regions of structural convergence. Building on this organization, scaffold decomposition, functional group analysis, and physicochemical property profiling are integrated to elucidate recurring E3 ligase-binding motifs, target-binding scaffolds, linker architectures, and PROTAC-specific property regimes that frequently extend beyond classical drug-likeness boundaries. By combining unsupervised clustering with interpretable chemical and property-based analyses, this work provides a systematic organization of PROTAC chemical space based on structural and physicochemical features. Collectively, this framework establishes generalizable principles for scaffold modification, linker optimization, and modular recombination, offering a rational foundation for lead optimization and the development of next-generation PROTACs with improved potency, selectivity, and developability.

## Methodology

### Data collection and preparation

**Fig. 2:**
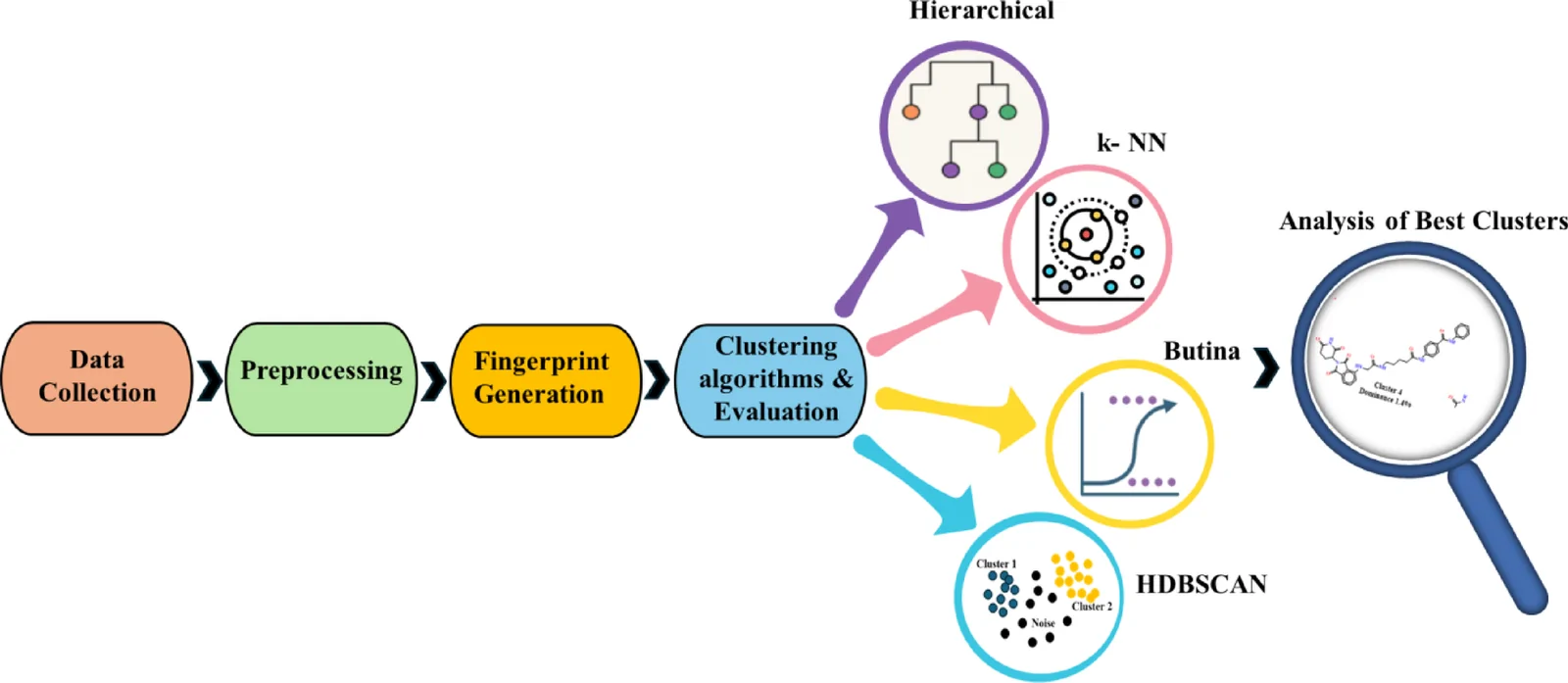
Computational workflow used in the current study: mapping the PROTAC chemical space using an unsupervised machine learning framework.

PROTAC molecules were retrieved from the PROTAC-DB database (https://protacdb.com/*).* The latest version, PROTAC-DB 3.0, provides structural information, SMILES (Simplified Molecular Input Line Entry System) representations, and UniProt identifiers for reported PROTAC compounds^32^. SMILES is a widely used linear notation that encodes molecular structures as character strings, enabling efficient storage, comparison, and computational analysis of chemical structures^33^. All available entries were downloaded in CSV format and used as the initial dataset for further processing^34^. An overview of the complete data curation and analysis pipeline is provided in Fig. 2.

### Data preprocessing and standardization

The dataset was processed using a standardized workflow implemented in Python. An initial set of 9,380 PROTAC entries was retrieved, all of which contained non-empty SMILES representations. Duplicate molecules were removed based on SMILES identity, resulting in 6,113 unique compounds. Molecular standardization was performed using the RDKit MolStandardize module, including largest-fragment selection, charge neutralization, and canonical SMILES generation. Molecules that could not be successfully standardized were discarded. The resulting 6,113 standardized SMILES were subsequently converted into RDKit molecular objects for downstream analysis^35,36^.

### Molecular fingerprint generation and dimensionality reduction

To reduce the number of bits, which originally consist of 2048-bit feature vectors, Principal Component Analysis (PCA) was applied using the scikit-learn library^37^. Dimensionality reduction was necessary to remove redundancy, reduce fingerprint noise, facilitate efficient similarity-based clustering, and visualize the chemical space^38^. PCA was selected because it is a deterministic, linear method that preserves global variance structure and enables reproducible and interpretable transformations of high-dimensional data, which are desirable properties for subsequent clustering analyses. PCA was applied to the 2048-dimensional fingerprint vectors, retaining the top 50 principal components based on explained variance, with a fixed random seed to ensure reproducibility. Clustering and exploratory analyses were conducted using these 50 principal components. Intra-cluster chemical similarity was evaluated using Tanimoto similarity computed on the original 2048-bit fingerprint representations^39^.

### Clustering algorithms and parameter optimization

Multiple unsupervised clustering algorithms were applied to PROTACs using Morgan fingerprint–based molecular representations, and their parameters were systematically optimized to group molecules according to structural similarity, with meaningful molecular groupings defined as clusters exhibiting high chemical similarity and shared scaffold features.

#### K-Means clustering

K-Means clustering was performed using the *KMeans* implementation from the scikit-learn library. The number of clusters (k) was explored in the range of 2 to 10. For each value of k, clustering was repeated with multiple random initializations (*n_init = 10*) and a fixed random seed to ensure reproducibility^40^. The algorithm partitions data by minimizing the within-cluster sum of squares, defined as:

$$\:arg\_min\sum\:_{i=1}^{k}\sum\:_{x\in\:\:Ci}{\left|\right|\:x\:-\:{\mu\:}_{i}\left|\right|}^{2}$$

#### Hierarchical clustering

Hierarchical clustering was performed using the agglomerative clustering approach implemented in the scikit-learn library. The number of clusters was evaluated from 2 to 10, and three clusters were selected for the final analysis based on internal evaluation metrics. Ward’s linkage method was used, which minimizes the increase in within-cluster variance at each step of the clustering process^41^.

$$\:{\Delta\:}E\:=\:\sum\:_{x\:\in\:{C}_{ab}}{\left(x\:-\:{\mu\:}_{ab}\right)}^{2}\sum\:_{x\:\in\:{C}_{a}}{\left(x\:-\:{\mu\:}_{a}\right)}^{2}\sum\:_{x\:\in\:{C}_{b}}{\left(x\:-\:{\mu\:}_{b}\right)}^{2}$$

#### HDBSCAN

Density-based clustering was performed using *hdbscan.HDBSCAN* on PCA-reduced data. The Euclidean distance metric was used. The *min_cluster_size* parameter was systematically varied, while *min_samples* was held fixed to control the cluster density requirements^42^. HDBSCAN defines clusters based on mutual reachability distance:

$$\:{d}_{mreach}\left(a,b\right)=max\left(cor{e}_{k}\left(a\right),cor{e}_{k}\left(b\right),d\left(a,b\right)\right)$$

#### Butina clustering

Similarity-based clustering was performed using RDKit’s *Butina.ClusterData* algorithm directly on Morgan fingerprints. Pairwise molecular distances were computed as:

$$\:{d}_{ij}=1-{T}_{ij}$$

Where $\:{T}_{ij}$ is the Tanimoto similarity? Multiple similarity cutoff values were evaluated to assess their effect on cluster formation^43^.

#### Cluster evaluation metrics

Clustering performance was evaluated using a combination of internal validation metrics and chemically interpretable scores to assess both geometric separation and chemical coherence of clusters. For K Means, Hierarchical Clustering, and HDBSCAN, cluster compactness and separation were quantified using the Silhouette Score, computed with sklearn.metrics.silhouette_score and defined as:

$$\:S=\frac{b-a}{max\left(a,b\right)}$$

where * a* is the mean intra-cluster distance and * b* is the mean distance to the nearest neighbouring cluster. This metric was not directly applicable to Butina clustering, as Butina employs a similarity-threshold-based assignment rather than centroid- or distance-optimized partitioning^44^.

Chemical homogeneity within clusters was quantified using mean intra-cluster Tanimoto similarity^45^, calculated via a custom implementation as

$$\:{T}_{ij}=\frac{c}{a+b-c}$$

where $\:{T}_{ij}$denotes the Tanimoto similarity between molecular fingerprints * i* and *j* within a cluster of size * n*.

Structural consistency was further evaluated using scaffold coherence, implemented as a custom metric and defined as

$$\:Scaffold\:Coherence=\frac{{N}_{dominant\:scaffold}}{{N}_{cluster}}$$

where $\:{N}_{dominant\:scaffold}$ represents the number of molecules sharing the most frequent Murcko scaffold within a cluster, identified using Murcko Scaffold, MurckoScaffoldSmiles, and $\:{N}_{cluster}$ is the total number of molecules in the cluster^46,47^.

Finally, cluster statistics, including the total number of clusters and the minimum, median, and maximum cluster sizes, were reported for each clustering method to characterize cluster granularity, size distribution, and the relative tendency of different algorithms, particularly Butina and HDBSCAN, to generate large coherent clusters versus fragmented or noise-dominated groupings^48^.

### In-depth characterization of top Butina clusters

The ten largest clusters obtained from Butina clustering using Tanimoto similarity^49^ were subjected to detailed chemical characterization. Bemis–Murcko scaffolds^50^, defined as core ring systems and linkers obtained by removing side chains, were identified within each cluster, as they provide a chemically intuitive representation of core molecular frameworks. A frequent Murcko scaffold was defined as the most frequently occurring scaffold in a given cluster, representing the shared core structure of the largest fraction of molecules, and scaffold dominance was quantified as the fraction of molecules sharing this core; representative scaffolds were visualized accordingly. Functional group analysis was performed using a curated set of 27 predefined functional groups, detected using SMARTS-based patterns^51^, and cluster-wise frequencies were visualized as heatmaps. Physicochemical properties were calculated using standard molecular descriptors, and their distributions across clusters were analyzed using comparative box plots and kernel density plots^52^.

### Visualization of chemical space

Chemical space and cluster separation were visualized using both linear and non-linear dimensionality reduction techniques. Here, chemical space refers to the abstract, high-dimensional representation of molecules based on their structural features, whereas the visualization of chemical space is its low-dimensional projection for interpretation. Such visualizations depend not only on the visualization technique but also, critically, on the underlying molecular representation, in line with the chemical multiverse concept^53^. PCA was applied to molecular fingerprint representations, and scatter plots of the first two principal components were generated with points colored by cluster assignment. In parallel, Uniform Manifold Approximation and Projection (UMAP; https://umap-learn.readthedocs.io) was employed to obtain nonlinear embeddings that complement PCA-based views^54^. Representative molecules and frequent scaffolds, defined as the most frequently occurring core frameworks within a cluster, were visualized using RDKit’s Draw. MolsToGridImage, while cluster size distributions, clustering evaluation metrics, and functional group prevalences were summarized using bar plots and heatmaps implemented with Seaborn and Matplotlib (https://matplotlib.org)^55^.

## Results and discussion

### Dataset curation and chemical space characterization

Systematic curation of the PROTAC-DB 3.0 dataset yielded a refined collection of 6,113 unique, chemically valid PROTAC molecules from an initial set of 9,380 entries. The exclusion of records with missing SMILES representations, structural redundancy, and non-standardizable molecules substantially improved dataset consistency and reliability. Importantly, the curated dataset retains the intrinsic modular diversity reported in PROTAC-DB, which includes 569 target-binding warheads, 107 E3 ligase ligands, and 5,753 linker architectures. This composition reflects the design flexibility of PROTACs, in which diverse warhead chemistries are combined with a comparatively small set of E3 ligase binders across a wide range of linker lengths and chemotypes.

PCA analysis based on molecular fingerprints revealed a broadly distributed and heterogeneous chemical space. The dispersion observed in the reduced-dimensional representation suggests that variations in linker composition and length, together with differences in warhead and E3 ligase ligand chemistry, collectively drive structural diversity among PROTACs. Such heterogeneity is characteristic of bifunctional molecules and distinguishes the PROTAC chemical space from that of conventional small-molecule drugs^56^. The resulting 50-dimensional principal component space provides a meaningful framework for identifying structurally related subsets, enabling subsequent clustering and comparative analyses. Overall, these results highlight the chemically diverse landscape of the curated PROTAC dataset, which has direct implications for rational PROTAC design and optimization.

### Clustering analysis and structural heterogeneity in PROTAC chemical space

The structural organization of the curated PROTAC dataset was systematically examined using multiple unsupervised clustering strategies, including K-Means, Hierarchical clustering, HDBSCAN, and Butina similarity-based clustering. The final configurations for each method were selected after extensive parameter exploration and are summarized in Table 1, which reports the number of clusters and key cluster size statistics. Together, these metrics provide a quantitative basis for comparing how different algorithms partition PROTAC chemical space at varying levels of resolution. As shown in Table 1, K-Means clustering with *k = 2* resulted in a highly coarse partitioning of the dataset into two large clusters, with sizes of 1,925 and 4,188 molecules and a median cluster size of 3,056.5. A detailed summary of clustering behaviour across k values is provided in the Supplementary Information (Table S1). This strong imbalance reflects the dominance of global structural variance in the PCA-reduced fingerprint space. It suggests that K-Means primarily captures broad molecular distinctions rather than fine-grained chemical similarities. Such behaviour is typical of centroid-based methods when applied to chemically diverse datasets such as PROTACs, where linker length, molecular weight, and overall topology contribute strongly to variance. Hierarchical clustering with *k = 3* produced a more balanced partitioning, yielding three clusters with sizes ranging from 1,720 to 2,493 molecules and a median cluster size of 1,900. Compared to K-Means, this method provides improved resolution while maintaining relatively uniform cluster sizes. The balanced nature of these clusters indicates that hierarchical clustering is better suited to capturing intermediate-level structural relationships within the PROTAC dataset, likely reflecting shared linker architectures or related warhead–E3 ligand combinations.

**Table 1:** Summary of clustering outcomes for selected methods, including the number of clusters and cluster size statistics.

S/*N*	Method	Num clusters	Min cluster size	Median cluster size	Max cluster size
0	kmeans_k2	2	1925	3056.5	4188
1	Hierarchical_k3	3	1720	1900.0	2493
2	HDBSCAN_min30	3	44	51.0	6010
3	Butina_cutoff0.5	403	1	5.0	751

Centroid-based clustering using K-means partitioned the dataset into two large clusters, with a minimum cluster size of 1,925 molecules, a median size of 3,056.5, and a maximum size of 4,188, indicating coarse segmentation. Similarly, hierarchical clustering produced three large clusters, with sizes ranging from 1,720 to 2,493 molecules and a median cluster size of 1,900, again reflecting broad partitioning. Density-based clustering using HDBSCAN resulted in three clusters with highly uneven distributions, with a minimum cluster size of 44 molecules, a median size of 51, and a frequent cluster containing 6,010 molecules. In contrast, Butina clustering generated 403 clusters, with a minimum cluster size of one molecule and a median cluster size of five. Although the largest cluster contained 751 molecules, the low median cluster size indicates substantially finer partitioning compared to the other clustering approaches.

**Fig. 3:**
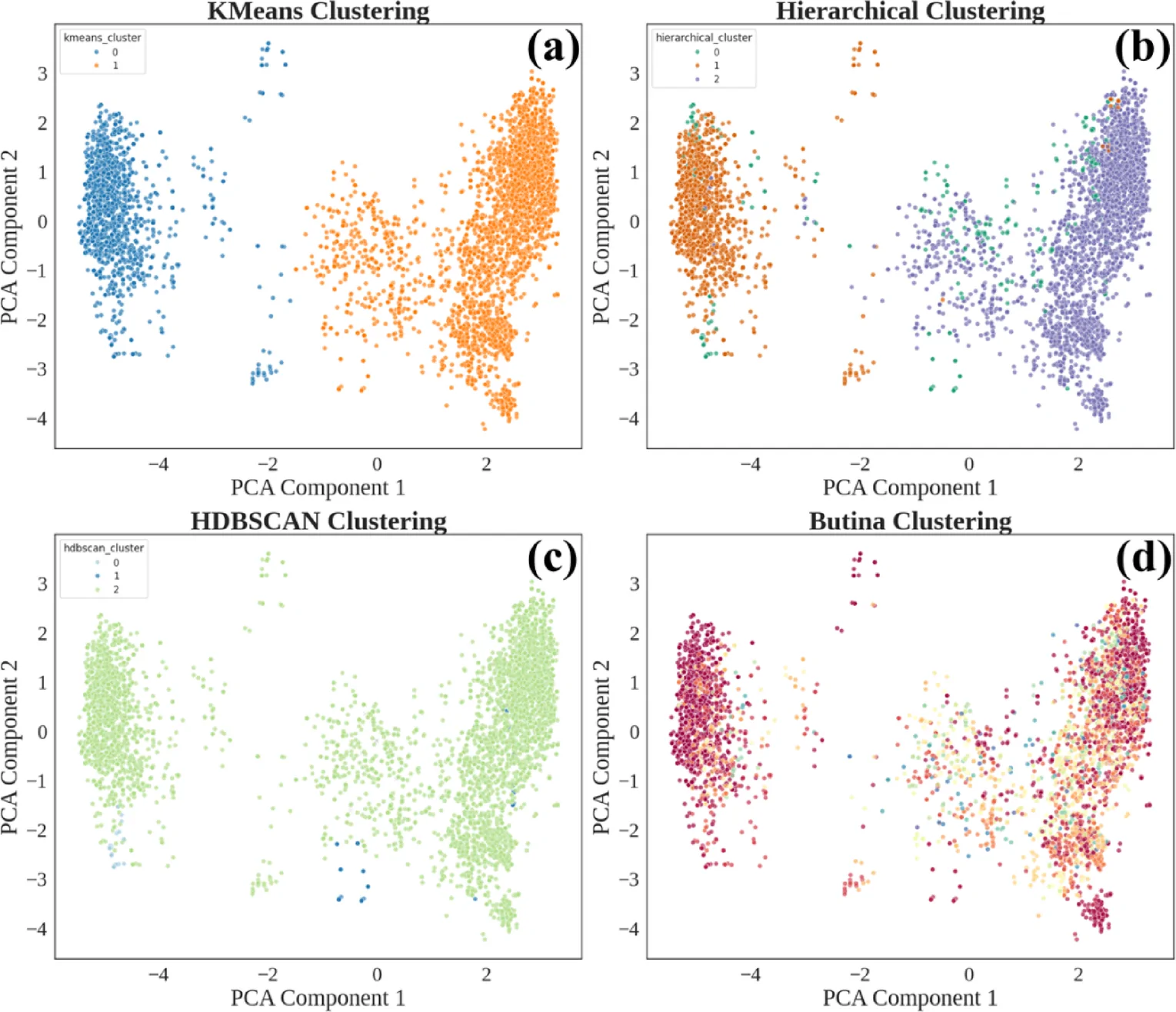
PCA-based visualization of PROTAC clusters obtained using (**a**) K-Means (2 clusters), (**b**) Hierarchical (3 clusters), (c) HDBSCAN (3 clusters), and (d) Butina clustering (403 clusters). Each colour represents a distinct cluster.

The impact of these clustering strategies on chemical space partitioning is visually illustrated in the PCA projections shown in Fig. 3. K-Means clustering produces a clear binary separation along the first principal component, reflecting frequent global variance but limited sensitivity to subtle structural differences. Hierarchical clustering subdivides this space into three partially overlapping yet distinct regions, suggesting improved discrimination of intermediate molecular classes. HDBSCAN identifies dense molecular cores within the same PCA landscape, while grouping the majority of molecules into a single large cluster, consistent with its density-driven design. In contrast, Butina clustering overlays a large number of small, interspersed clusters across the PCA space, highlighting chemically coherent groups that are not necessarily separable along global variance axes.

**Fig. 4:**
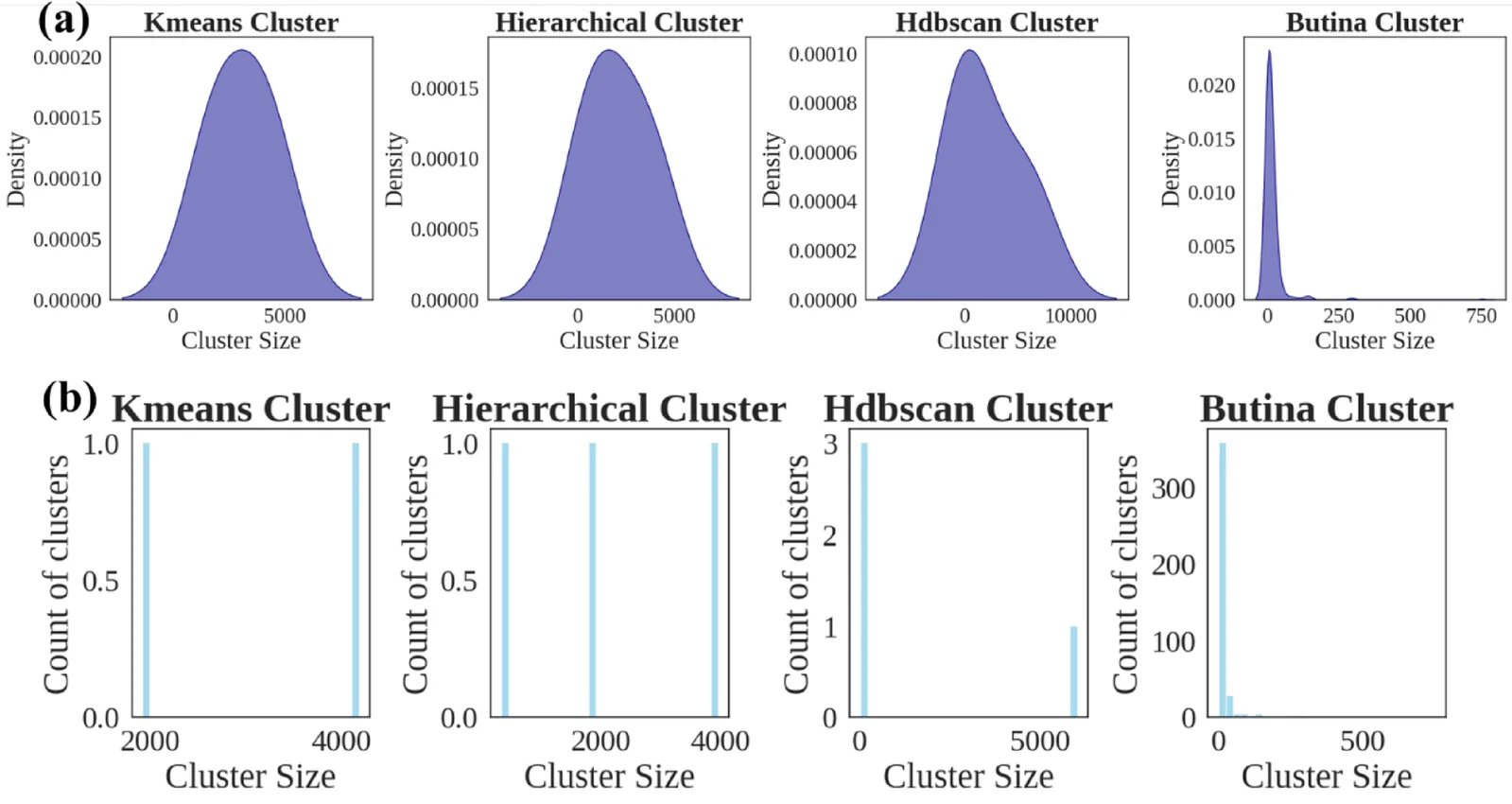
Cluster size distributions obtained using different clustering approaches: (**a**) kernel density estimates illustrating the overall distribution of cluster sizes, and (**b**) histograms depicting the frequency of clusters across size ranges for the selected methods.

Further insights into cluster granularity and size variability are provided by the cluster size histograms and density plots shown in Figs. 4**(a and b)**. For K-Means and Hierarchical clustering, the distributions are dominated by a small number of large clusters, resulting in smooth, unimodal density profiles. These patterns indicate low granularity and strong aggregation of molecular structures. HDBSCAN exhibits a mixed distribution, characterized by one frequent cluster and a small number of compact clusters, reflecting uneven density across chemical space. In contrast, Butina clustering shows a sharply skewed distribution with a pronounced peak at small cluster sizes and a long tail extending toward larger clusters, a hallmark of similarity-based clustering applied to chemically diverse datasets.

The density plots further emphasize these differences by illustrating how cluster sizes are distributed across methods. The narrow, smooth density profiles observed with centroid-based methods contrast sharply with the highly peaked distribution observed with Butina clustering, underscoring its emphasis on local chemical similarity. HDBSCAN occupies an intermediate position, capturing both dense molecular neighbourhoods and broader structural continuity. Overall, these results demonstrate that PROTAC chemical space exhibits both strong global organization and extensive local structural diversity. Centroid-based methods effectively capture frequent molecular trends; hierarchical clustering offers balanced intermediate resolution, density-based methods highlight dense design families; and similarity-based clustering reveals fine-scale scaffold heterogeneity. The complementary insights obtained from these approaches underscore the importance of employing multiple clustering paradigms for comprehensive analysis of PROTAC molecular diversity.

**Fig. 5:**
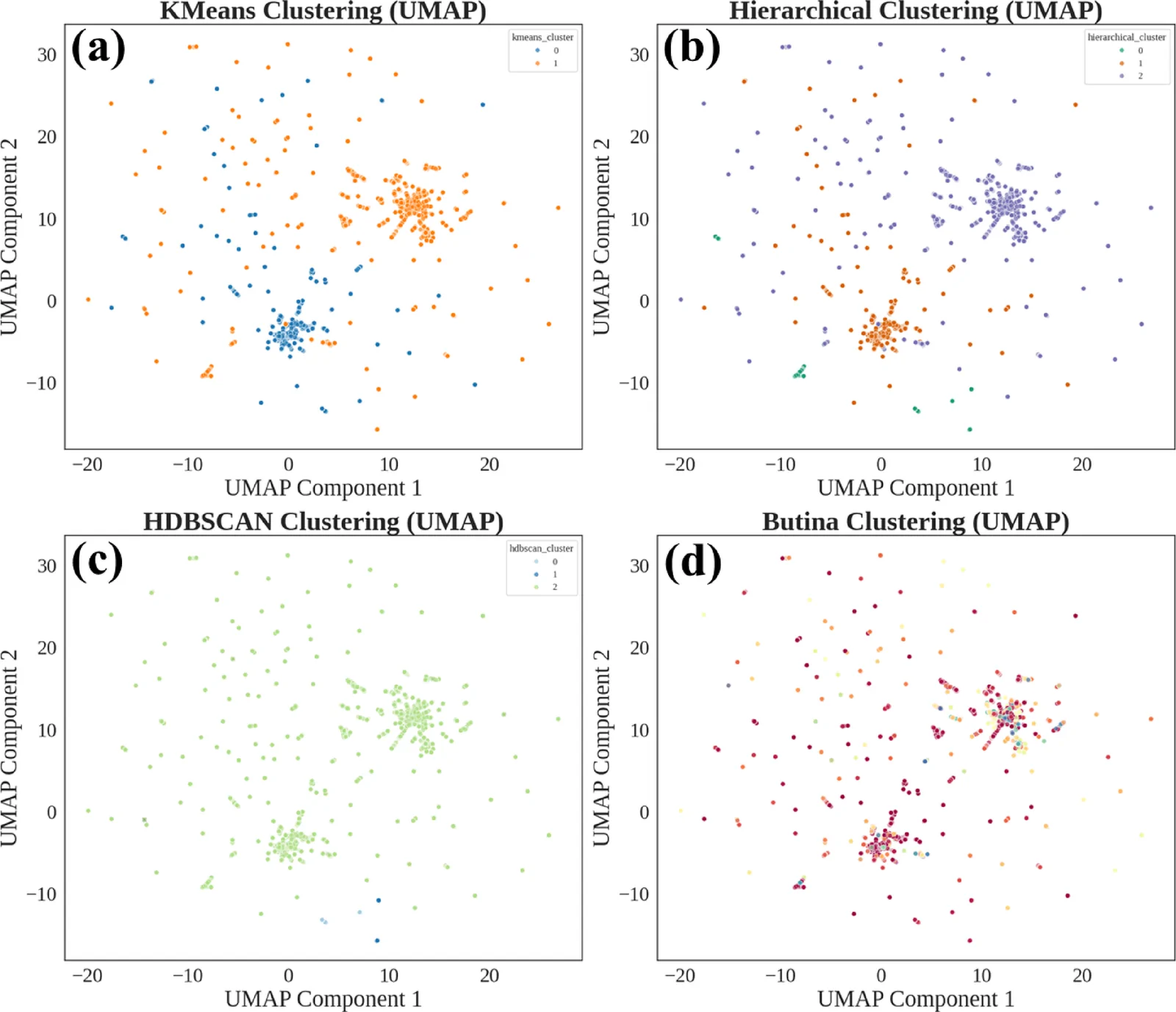
UMAP projections of PROTAC molecules colored by cluster assignments obtained using (**a**) K-Means, (**b**) Hierarchical, (**c**) HDBSCAN, and (**d**) Butina clustering.

To further investigate the local neighbourhood structure and non-linear relationships within the PROTAC chemical space, Uniform Manifold Approximation and Projection (UMAP) was employed to visualize clustering results obtained from different algorithms (Fig. 4). Unlike PCA, which primarily captures global variance, UMAP preserves local similarities and is therefore particularly suited for assessing how effectively clustering methods identify compact and chemically meaningful molecular neighbourhoods. In the UMAP projection for K-Means clustering (Fig. 5A), the two clusters appear as moderately separated but spatially overlapping groups. While dense cores are evident within each cluster, substantial intermixing is observed at the cluster boundaries. This behaviour reflects the centroid-based nature of K-Means, which assigns molecules based on their proximity to global centroids rather than on local structural similarity. Although K-Means achieved moderate silhouette and intra-Tanimoto values, scaffold coherence scores remained low (0.005–0.023), indicating limited clustering consistency at the core scaffold level. Hierarchical clustering with three clusters (Fig. 5B) demonstrates improved local separation compared to K-Means. Distinct compact regions corresponding to individual clusters are observed, particularly for the two frequent clusters, while a third cluster appears more dispersed. This pattern suggests that hierarchical clustering benefits from its iterative merging strategy, allowing it to capture intermediate-scale structural relationships among PROTAC molecules. However, partial overlap among clusters indicates that hierarchical clustering still prioritizes global structure over fine-grained local similarity. The UMAP representation of HDBSCAN clustering (Fig. 5C) reveals a markedly different organization. Dense molecular cores are clearly visible, surrounded by more sparsely populated regions. The majority of molecules are assigned to a frequent dense cluster, while smaller clusters and isolated points represent localized high-density regions or structurally distinct chemotypes. This visualization highlights HDBSCAN’s strength in identifying dense neighbourhoods within the PROTAC chemical space, as well as its ability to separate structurally ambiguous molecules that do not belong to well-defined dense regions.

In contrast, Butina clustering at a Tanimoto cutoff of 0.5 (Fig. 5D) results in extensive fragmentation across the UMAP space, with numerous small, tightly packed clusters dispersed throughout the embedding. Each cluster corresponds to a group of molecules with high pairwise similarity, often reflecting shared core scaffolds or closely related linker architectures. Although clusters appear spatially intermingled globally, local compactness within them is pronounced, underscoring the scaffold-driven nature of Butina clustering. This behaviour is particularly advantageous for identifying chemically homogeneous subsets within the highly diverse PROTAC dataset.

Collectively, the UMAP visualizations corroborate trends observed in PCA-based analyses while providing enhanced insight into local structural relationships. Centroid-based methods emphasize global partitioning, density-based clustering highlights dense molecular neighbourhoods, and similarity-based clustering captures fine-scale scaffold organization. These complementary perspectives demonstrate that PROTAC chemical space is characterized by both broad structural trends and highly localized similarity patterns, reinforcing the need for multi-method clustering strategies in PROTAC cheminformatics studies.

### Model evaluation and validation matrices

**Fig. 6:**
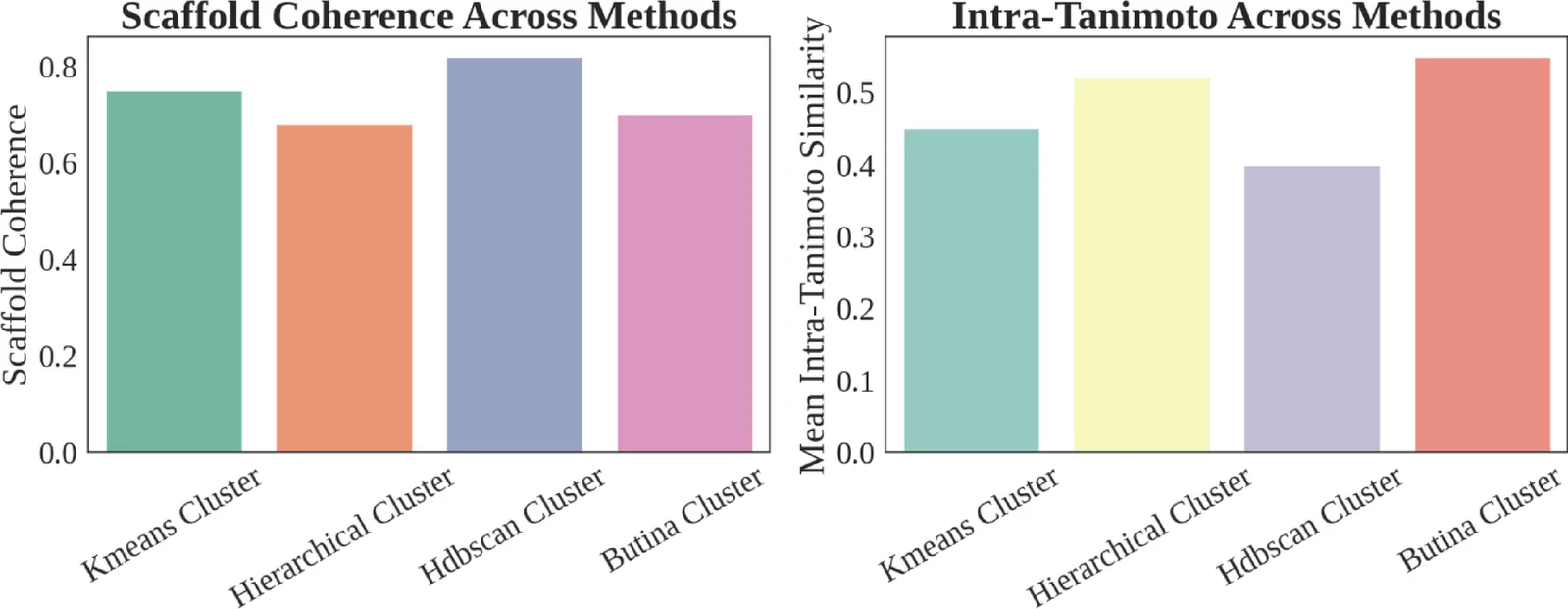
Comparative evaluation of clustering methods based on chemical validation metrics. (**A**) Scaffold coherence across clustering methods. (**B**) Mean intra-cluster Tanimoto similarity across clustering methods.

To comprehensively evaluate clustering performance, both geometric separation metrics and chemically interpretable validation metrics were employed. This dual evaluation strategy is particularly important for PROTAC datasets, where chemical relevance and scaffold consistency are often more critical than purely geometric separation in feature space. Quantitative results for all evaluated methods are summarized in Table 2, while a comparative visualization of key chemical metrics is presented in Fig. 6.

The Silhouette Score was used to assess cluster compactness and separation for centroid-based and density-based methods. Among the evaluated approaches, K-Means clustering (*k = 2*) achieved the highest Silhouette Score (0.258), indicating relatively strong global separation of clusters in the PCA-reduced fingerprint space. Hierarchical clustering (*k = 3*) yielded a lower Silhouette Score (0.158), reflecting increased overlap between clusters as the number of partitions increased. HDBSCAN (*min_cluster_size = 30*) exhibited a further reduction in Silhouette Score (0.146), which can be attributed to its density-driven design and the presence of highly imbalanced cluster sizes. These results suggest that centroid-based methods are more effective in capturing global geometric structure, but do not necessarily produce chemically homogeneous clusters.

To directly assess chemical similarity within clusters, mean Intra-Cluster Tanimoto similarity was calculated for all methods. As shown in Table 2 and visualized in Fig. 6, K-Means and Hierarchical clustering achieved relatively low Intra-Tanimoto values (0.326 and 0.317, respectively), indicating limited structural homogeneity within clusters. HDBSCAN demonstrated a marked improvement, achieving an Intra-Tanimoto similarity of 0.587, suggesting that density-based clustering more effectively groups structurally related PROTAC molecules within dense regions of chemical space. Notably, Butina clustering at a similarity cutoff of 0.5 achieved the highest Intra-Tanimoto similarity (0.748), highlighting its strong ability to form chemically coherent clusters based on explicit fingerprint similarity.

Scaffold coherence, defined as the fraction of molecules within a cluster sharing the frequent Murcko scaffold, provides an additional layer of chemical validation. As illustrated in Fig. 6A, scaffold coherence values for K-Means (0.005) and Hierarchical clustering (0.006) were negligible, indicating that these methods do not preserve scaffold-level relationships. HDBSCAN showed a moderate increase in scaffold coherence (0.030), reflecting partial scaffold enrichment within dense clusters. In contrast, Butina clustering exhibited a substantially higher scaffold coherence (0.385), demonstrating its effectiveness in organizing PROTAC molecules into scaffold-consistent groups. This behaviour is particularly relevant for PROTAC design, where linker-warhead-E3 ligand combinations often revolve around conserved core scaffolds. Although the Silhouette Score is not directly applicable to Butina clustering due to its similarity-threshold-based assignment and the frequent occurrence of small or single-member clusters, the chemical validation metrics provide a more appropriate evaluation framework. The combined evidence from Table 2; Fig. 6 clearly indicates that Butina clustering offers superior chemical interpretability, even if it does not optimize global geometric separation.

Overall, the comparative evaluation reveals a clear trade-off between geometric clustering quality and chemical relevance. Centroid-based methods emphasize global structure, density-based clustering captures dense molecular neighbourhoods, and similarity-based clustering maximizes scaffold and structural consistency. Based on its superior performance in Intra-Tanimoto similarity and Scaffold Coherence, Butina clustering with a Tanimoto cutoff of 0.5 was selected for detailed chemical space characterization and downstream PROTAC analysis.

**Table 2:** Comparison of clustering methods using geometric and chemical validation metrics, including silhouette score, intra-cluster tanimoto similarity, and scaffold coherence.

S/*N*	Method	Silhouette Score	Intra-Tanimoto	Scaffold coherence
0	kmeans_k2	0.258	0.326	0.005
1	Hierarchical_k3	0.158	0.317	0.006
2	HDBSCAN_min30	0.146	0.587	0.030
3	Butina_cutoff0.5	-	0.748	0.385

### Analysis of top Butina clusters

**Fig. 7:**
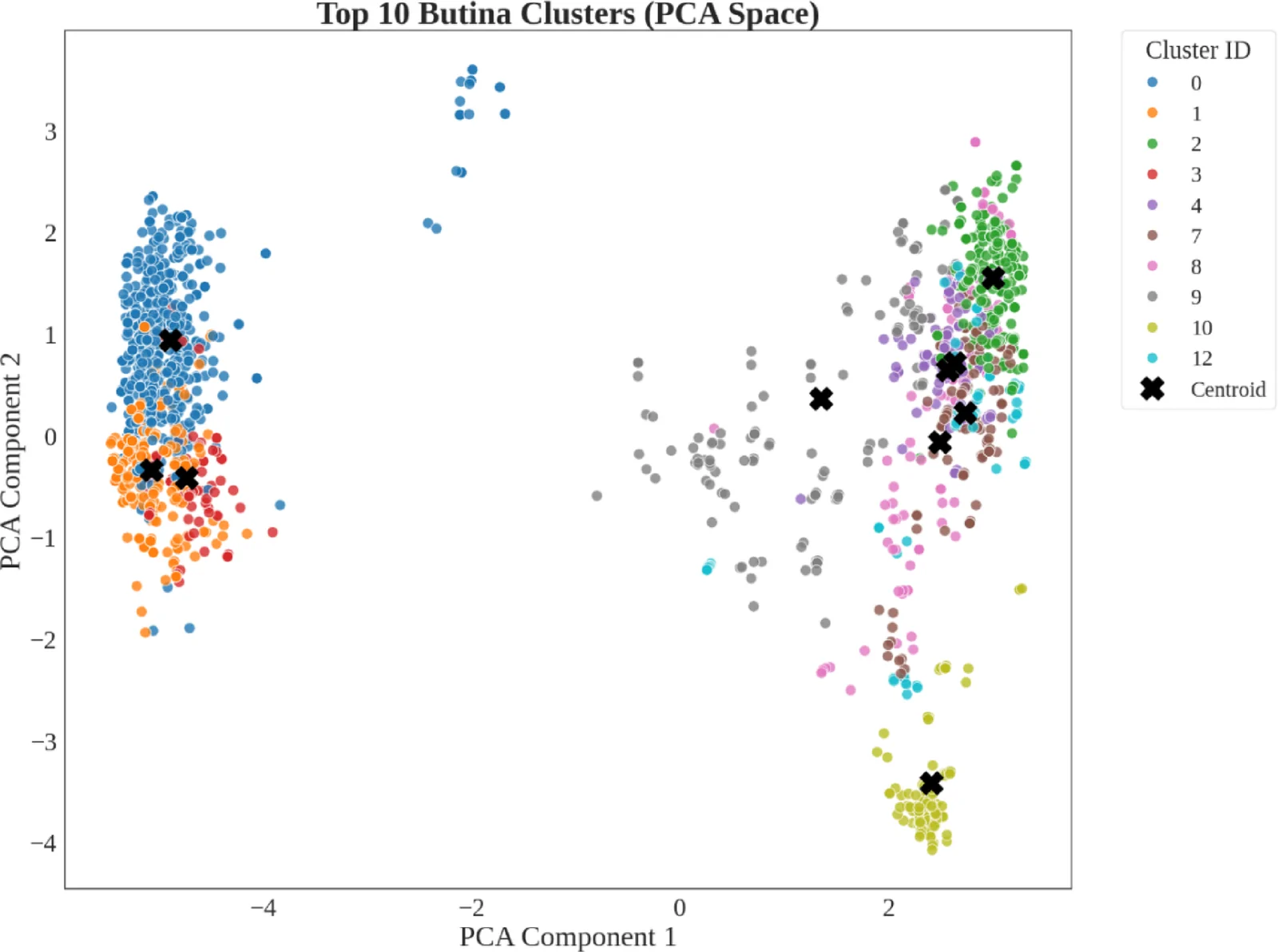
PCA-based visualization of the top 10 largest Butina clusters (Tanimoto cutoff = 0.5), showing cluster centroids and spatial separation in reduced chemical space.

Similarity-based Butina clustering with a Tanimoto cutoff of 0.5 identified 403 distinct molecular clusters, reflecting the high structural diversity of the PROTAC dataset. The resulting cluster size distribution was strongly right-skewed, characterized by a small number of large clusters and a large number of small clusters, including several single-member clusters. This pattern is typical for similarity-driven clustering applied to chemically heterogeneous libraries and indicates the coexistence of frequent structural motifs alongside numerous less-represented or unique chemical series. Among the 403 clusters, the 10 largest clusters were selected for detailed analysis due to their substantial contribution to the overall dataset composition and their potential relevance as recurring PROTAC design families. The spatial organization of these top clusters in PCA-reduced chemical space is illustrated in Fig. 7, where each cluster is represented along with its corresponding centroid. The visualization reveals that the largest clusters form compact, well-defined regions, indicating strong intra-cluster similarity, consistent with the high Intra-Tanimoto values observed for Butina clustering. The centroids of these clusters are clearly separated along the principal components, suggesting that each large cluster corresponds to a distinct structural family rather than overlapping chemical motifs.

Notably, clusters located on opposite ends of the first principal component exhibit pronounced separation, implying fundamental differences in molecular features such as linker length, molecular size, or overall topology. Within individual clusters, limited dispersion around the centroid is observed, further supporting the scaffold-level coherence achieved by similarity-based clustering. Smaller but still prominent clusters (e.g., Clusters 10 and 12) occupy more isolated regions of the PCA space, suggesting that these groups represent chemically distinct but less frequently explored PROTAC architectures.

Overall, the analysis of the top Butina clusters demonstrates a clear balance between structural convergence and chemical diversity within the dataset. Large clusters reflect frequent and repeatedly optimized PROTAC scaffolds, whereas the long tail of smaller clusters captures exploratory or niche chemical designs. The identification and visualization of these major clusters provide valuable insight into prevailing PROTAC design strategies and establish a rational basis for selecting representative clusters for further structure–activity relationship analysis and targeted optimization.

### E3 ligase and target protein distribution for top ten Butina clusters

To address the biological interpretability of similarity-based clustering in the context of PROTAC design, we integrated curated E3 ligase recruiter and target protein annotations from the PROTAC DB export (protac.csv) into the merged dataset used for fingerprint generation and Butina clustering. This enabled a direct assessment of whether the largest chemically coherent clusters are enriched for specific E3 ligase recruiters and whether they exhibit target-centric patterns, which are central design dimensions for PROTACs. As shown in Table 3, the top 5 Butina clusters represent a substantial fraction of the dataset and include Cluster 0 (751 molecules), Cluster 1 (296 molecules), Cluster 2 (295 molecules), Cluster 9 (145 molecules), and Cluster 4 (145 molecules). The complete recruiter and target distributions for all annotated clusters are provided in the Supplementary Information (Figures S1 and S2). The presence of multiple large clusters with comparable sizes is consistent with repeated exploration of recurring PROTAC scaffold and linker design families. In contrast, the progressive decrease in cluster size indicates increasing chemical specialization among less frequently sampled architectures.

Overlaying recruiter information revealed a clear E3 ligase-centric structure within the chemically defined clusters. Details of the E3 ligase distribution for each cluster are provided in the Supplementary Information (Figure S1). Two of the largest clusters were enriched for the von Hippel-Lindau E3 ligase recruiter (VHL): Cluster 0 contained 749 VHL recruiting molecules out of 751 total (with a minor cIAP1 presence of 2 molecules), and Cluster 1 was exclusively VHL recruiting (296 of 296). In contrast, Cluster 2 was enriched for the cereblon E3 ligase recruiter (CRBN) and contained 295 CRBN recruiting molecules out of 295. Several other high-occupancy clusters were similarly CRBN-enriched, including Cluster 4 (145 of 145), Cluster 7 (143 of 143), and Cluster 10 (138 of 138). Limited mixing of recruiter chemotypes was observed in a small number of clusters, including Cluster 8, which was predominantly CRBN (140 of 141) with a minor MDM2 subset (1 of 141), and Cluster 12, which was primarily CRBN (83 of 94) with a notable DCAF16 subset (11 of 94). Overall, these results indicate that similarity-based clustering often captures recruiter-specific chemical series, while also highlighting occasional overlap in which shared linker or warhead features contribute to global fingerprint similarity.

Target distributions provided complementary evidence of target-centric organization across several clusters. The VHL-enriched clusters displayed broad target coverage, consistent with the reuse of a common recruiter scaffold across diverse warheads; for example, Cluster 0 was associated with ER, AKT1, AR, WDR5, and HDAC1 among the most frequent targets, while Cluster 1 showed enrichment for ER and additional targets such as NAMPT, SMARCA2, NSD3, and SHP2. In contrast, several CRBN-enriched clusters showed pronounced enrichment for specific targets, consistent with focused design series. Cluster 9 was largely CRBN-recruiting (142 of 145) and was strongly centred on bromodomain targets, with BRD4 and BRD2 comprising the majority of annotations. Cluster 10 was AR-centric at the target level, with AR accounting for 136 of 138 molecules and AR T878A accounting for 2 of 138. Cluster 8 was BTK-centric, with BTK representing 115 of 141 molecules. Cluster 3 was VHL-enriched and showed enrichment for kinase targets, including ALK and EGFR L858R/T790M, as well as LRRK2, BCR ABL, and CDK4. Cluster 12, which included both CRBN and DCAF16 recruiters, was strongly enriched for PARP1 and PARP2 targets and also contained KRAS G12C, CDK9, and FLT3 ITD annotations.

Taken together, the combined recruiter and target overlays demonstrate that the largest Butina clusters are not only chemically coherent but also exhibit clear E3 ligase-centric and target-centric signatures. This directly improves the practical interpretability of the clustering framework by connecting dominant chemical neighbourhoods to recruiter selection and recurring target or warhead series, thereby supporting ligase- and target-informed navigation of PROTAC chemical space.

**Table 3:** E3 Ligase and target protein distribution for the top ten Butina clusters.

Cluster ID	Total molecules	Top Five E3 ligases		Top five target proteins	
		E3 ligases	Molecules	Target proteins	Molecules
0	751	VHL cIAP1	749 2	ER AKT1 AR WDR5 HDAC1	51 37 33 31 30
1	296	VHL	296	ER NAMPT SMARCA2 NSD3 SHP2	60 37 19 18 17
2	295	CRBN	295	HDAC8 PARP1 TYR STING Alpha-tubulin	34 21 14 10 10
9	145	CRBN DCAF16 RNF4 cIAP1	142 1 1 1	BRD4 BRD2 BRD4 BD1 BRD3 BRD3 BD1	122 17 4 1 1
4	145	CRBN	145	CDK2 CDK9 sEH STING HDAC6	17 17 9 9 9
7	143	CRBN	143	ALK Wee1 FAK CDK4 AURKA	46 32 30 9 8
8	141	CRBN MDM2	140 1	BTK GSK3A ERalpha IGF-1R PARP1	115 14 3 2 1
10	138	CRBN	138	AR AR T878A	136 2
3	118	VHL	118	ALK EGFR L858R/T790M LRRK2 BCR-ABL CDK4	25 18 16 13 8
12	94	CRBN DCAF16	83 11	PARP1 PARP2 KRAS G12C CDK9 FLT3-ITD	58 11 11 8 3

### Frequent Murcko scaffold and physicochemical analysis of top Butina clusters

**Fig. 8:**
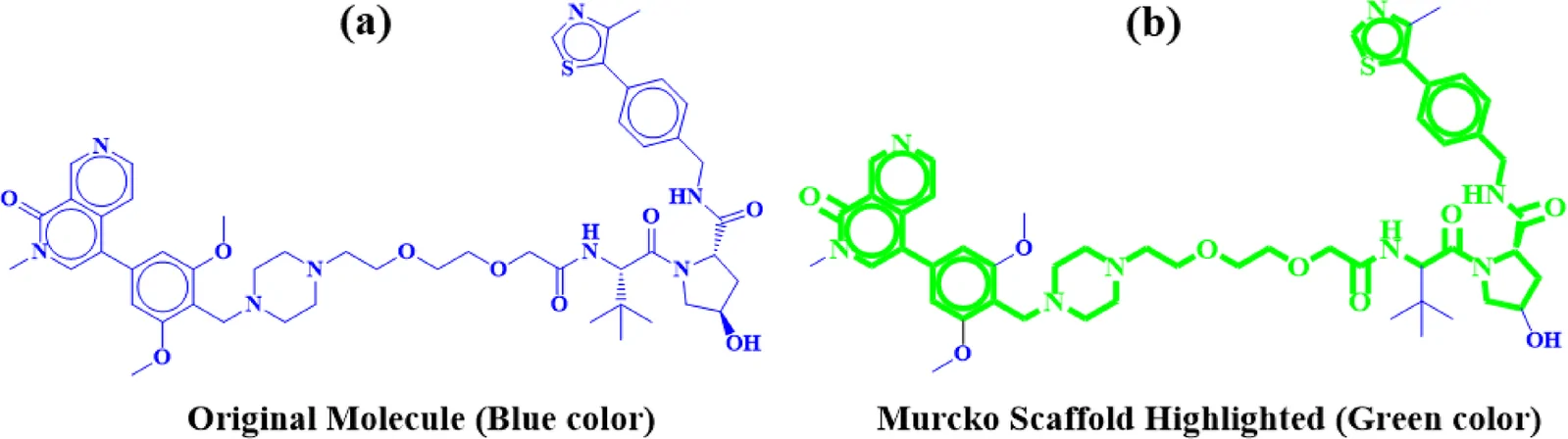
(**A**) Original chemical structure of the compound. (**B**) Murcko scaffold of the molecule, highlighted in green, representing the core ring framework after removal of side chains.

**Fig. 9:**
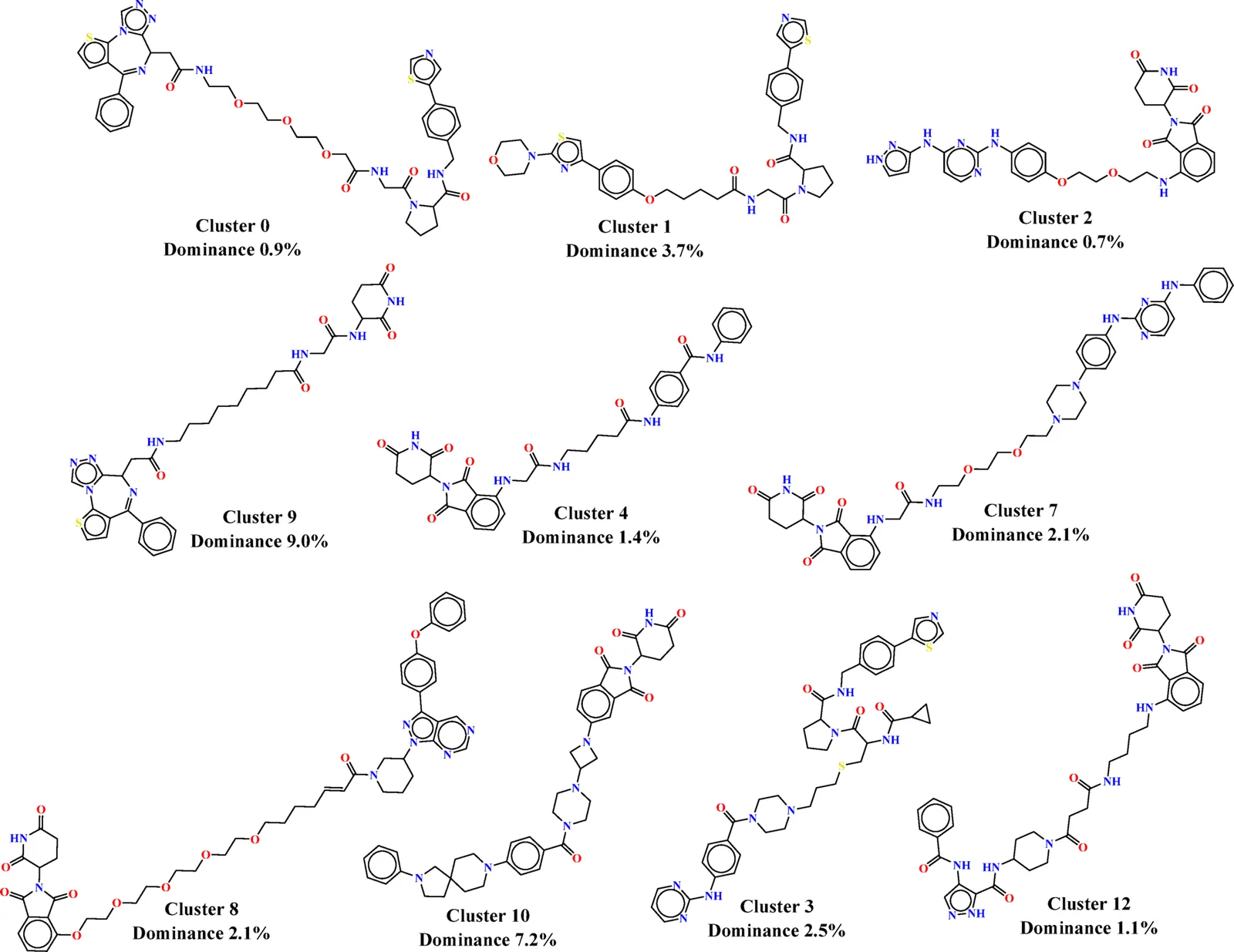
Top ten frequent clusters obtained from Butina clustering based on molecular fingerprint similarity. A representative compound is assigned to each cluster, and the percentage indicates the dominance of that cluster in the dataset.

For each of the top 10 Butina clusters (cutoff = 0.5), the most frequent Murcko scaffold was identified, and its dominance percentage was calculated to quantify scaffold conservation within major similarity-based groups (e.g., Fig. 8). The frequent scaffolds, visually presented to highlight core structural motifs, reveal chemically distinct yet predominantly PROTAC-like architectures characterized by bifunctional cores, extended linker systems, and recurring cyclic imide or amide functionalities associated with E3 ligase recruitment. The largest cluster (Cluster 0; dominance 0.93%) is centred on cyclic imide/amide scaffolds with complex side chains that terminate in urea or amide linkages, consistent with canonical PROTAC designs (Fig. 9). Cluster 1 (3.72%) is enriched in sulfur-containing heterocycles such as thiophene and thiazole combined with large hydrophobic regions and multiple amide bonds, suggesting a distinct PROTAC chemotype or alternative target–ligase pairing. Cluster 2 (0.68%) is dominated by fused heterocyclic systems, frequently quinazoline-based, attached to long PEG-like linkers and terminating in cyclic imides, consistent with kinase-targeting PROTACs incorporating thalidomide-like E3 ligase binders. Higher scaffold dominance in Clusters 9 (8.97%) and 10 (7.25%) reflects strong structural convergence, with benzothiazole-based bicyclic cores and spirocyclic or polycyclic frameworks, respectively, likely optimized for specific binding pockets or improved pharmacokinetic properties. The remaining clusters (Clusters 3, 4, 7, 8, and 12) exhibit moderate scaffold dominance (≈ 1–3%) and encompass benzodiazepinone, naphthyridine, triazole- or imidazole-rich, quinoline/isoquinoline, and cyclic amine-centred frameworks, collectively illustrating the breadth of PROTAC scaffold diversity.

**Fig. 10:**
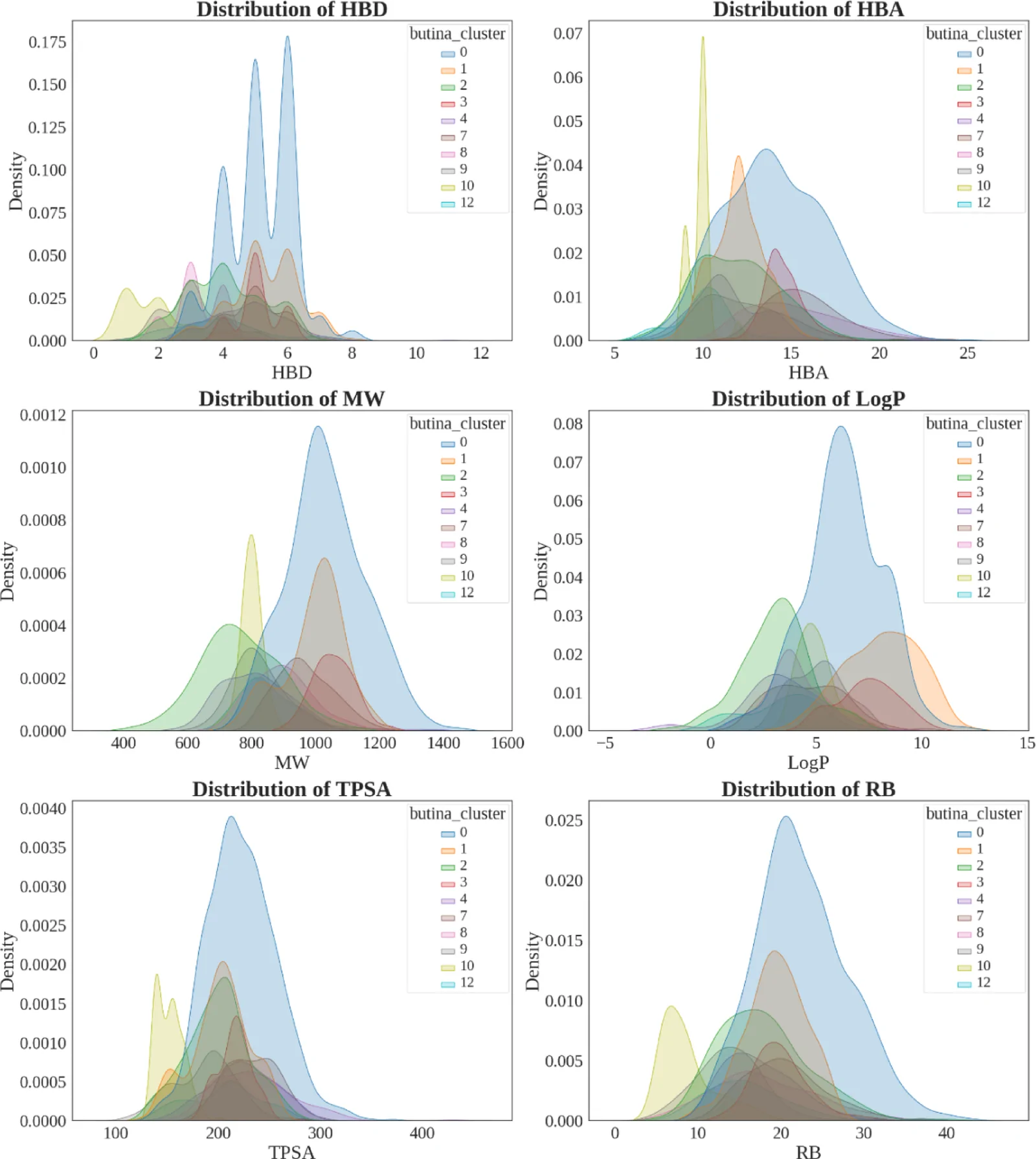
Kernel density distributions of six physicochemical properties (HBD, HBA, MW, LogP, TPSA, and RB) across the top 10 Butina clusters. Each colored curve represents a different cluster obtained using the Butina unsupervised clustering algorithm based on molecular similarity. Colours denote cluster IDs as shown in the legend.

**Fig. 11:**
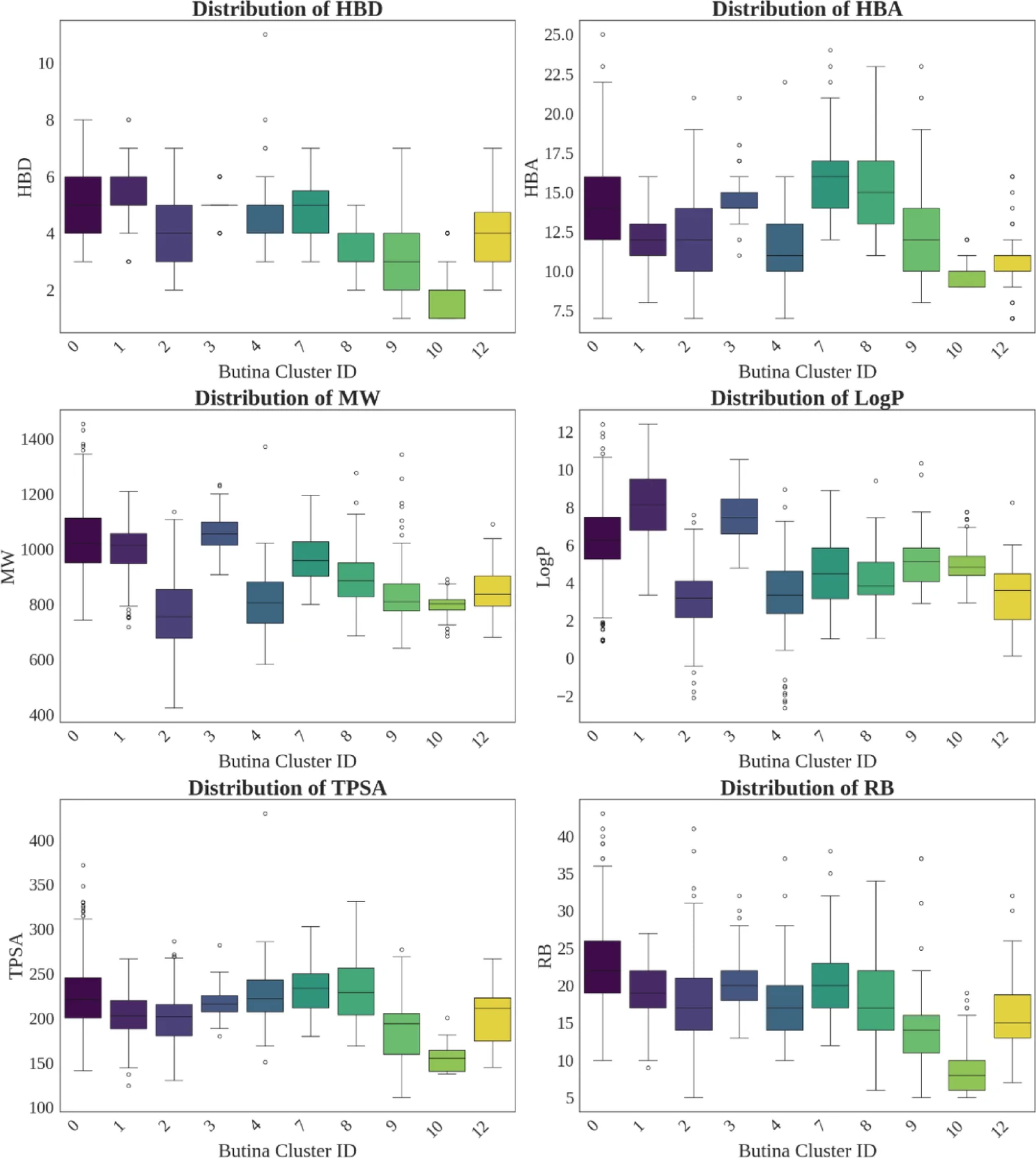
Box‑plot distributions of six physicochemical properties (HBD, HBA, MW, LogP, TPSA, and RB) across the top 10 Butina clusters. Each box represents a different cluster obtained using the Butina unsupervised clustering algorithm based on molecular similarity. Colours denote cluster IDs as shown in the legend.

Complementary physicochemical profiling of these clusters was performed using six key properties: molecular weight (MW), lipophilicity (LogP), topological polar surface area (TPSA), hydrogen‑bond donors (HBD), hydrogen‑bond acceptors (HBA), and rotatable bonds (RB), with distributions visualized through KDE plots and box plots (Figs. 10 and 11). Across all clusters, MW distributions are markedly shifted toward high values, typically exceeding 600–700 Da, reflecting the bifunctional nature of PROTACs and their requirement for multivalent interactions despite frequent violations of traditional Rule-of-Five criteria. LogP values are generally moderate to high (often > 3–4), indicating substantial lipophilicity necessary for cellular permeability, while simultaneously highlighting the challenge of balancing solubility and membrane transport. TPSA values are consistently elevated (typically > 100–150 Å²), driven by abundant amide, amine, and ether functionalities, thereby supporting aqueous solubility but potentially limiting passive diffusion. Correspondingly, HBD counts are moderate to high (typically 3–10+), and HBA counts are notably high (often 8–20+), enabling extensive hydrogen‑bonding interactions with protein targets that are crucial for ternary complex stabilization. High RB counts (frequently 10–30+) across clusters further underscore the pronounced conformational flexibility of PROTAC molecules, particularly within linker regions, which is essential for accommodating diverse target–E3 ligase geometries. Importantly, the KDE and box‑plot visualizations reveal clear intercluster differences in these property distributions, confirming that Butina clustering effectively segregates molecules into chemically coherent groups with distinct physicochemical profiles.

Overall, the integrated Murcko scaffold and physicochemical analyses demonstrate that the largest Butina clusters correspond to well‑defined PROTAC design families, each characterized by specific core scaffolds and property signatures. The observed property ranges align closely with the known PROTAC design space and reinforce the necessity of PROTAC‑specific optimization strategies, rather than reliance on conventional small‑molecule drug‑likeness criteria.

### Functional group analysis

**Fig. 12:**
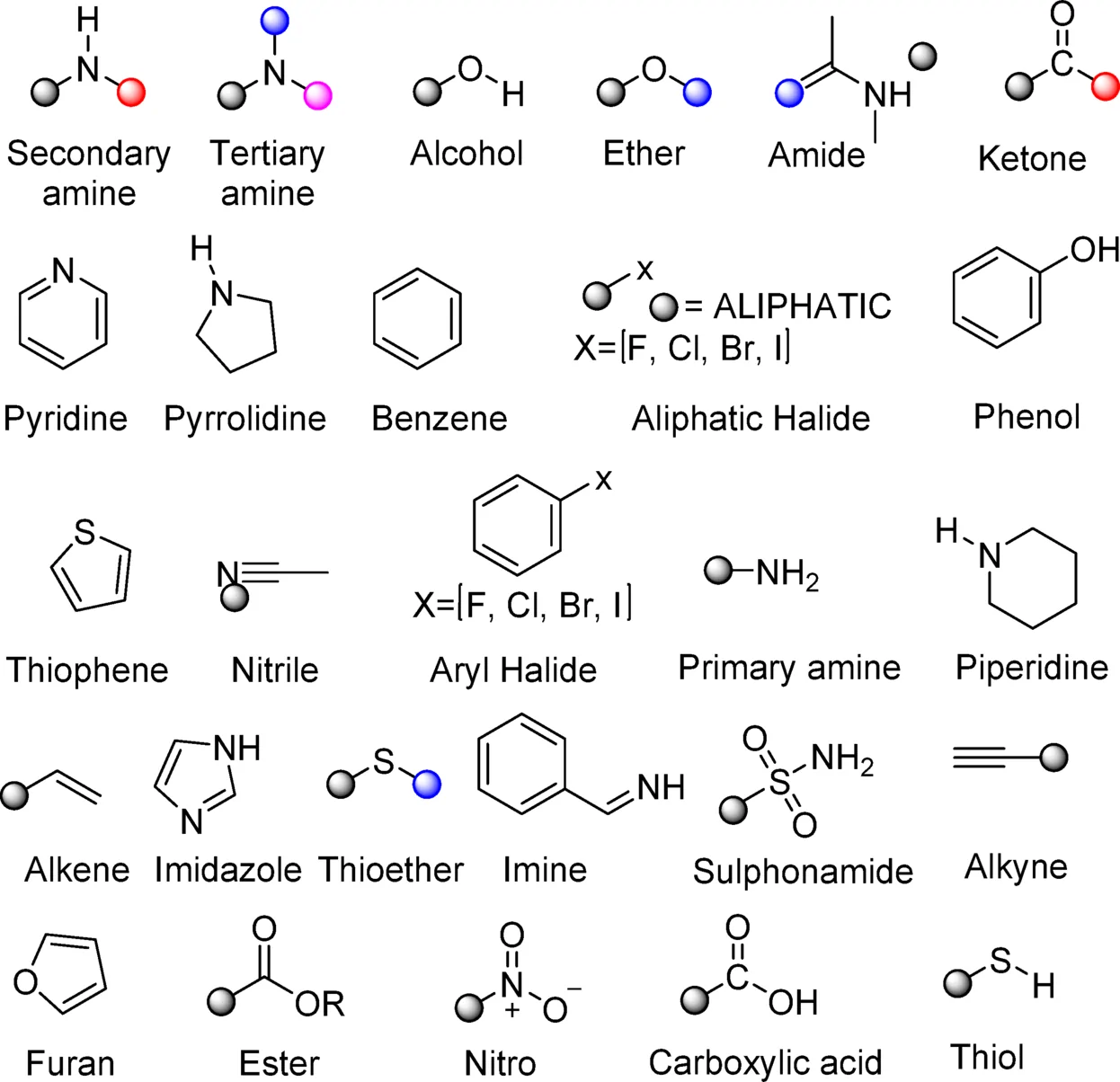
Representative structures of the 27 functional groups used for SMARTS-based detection in the PROTAC dataset.

**Fig. 13:**
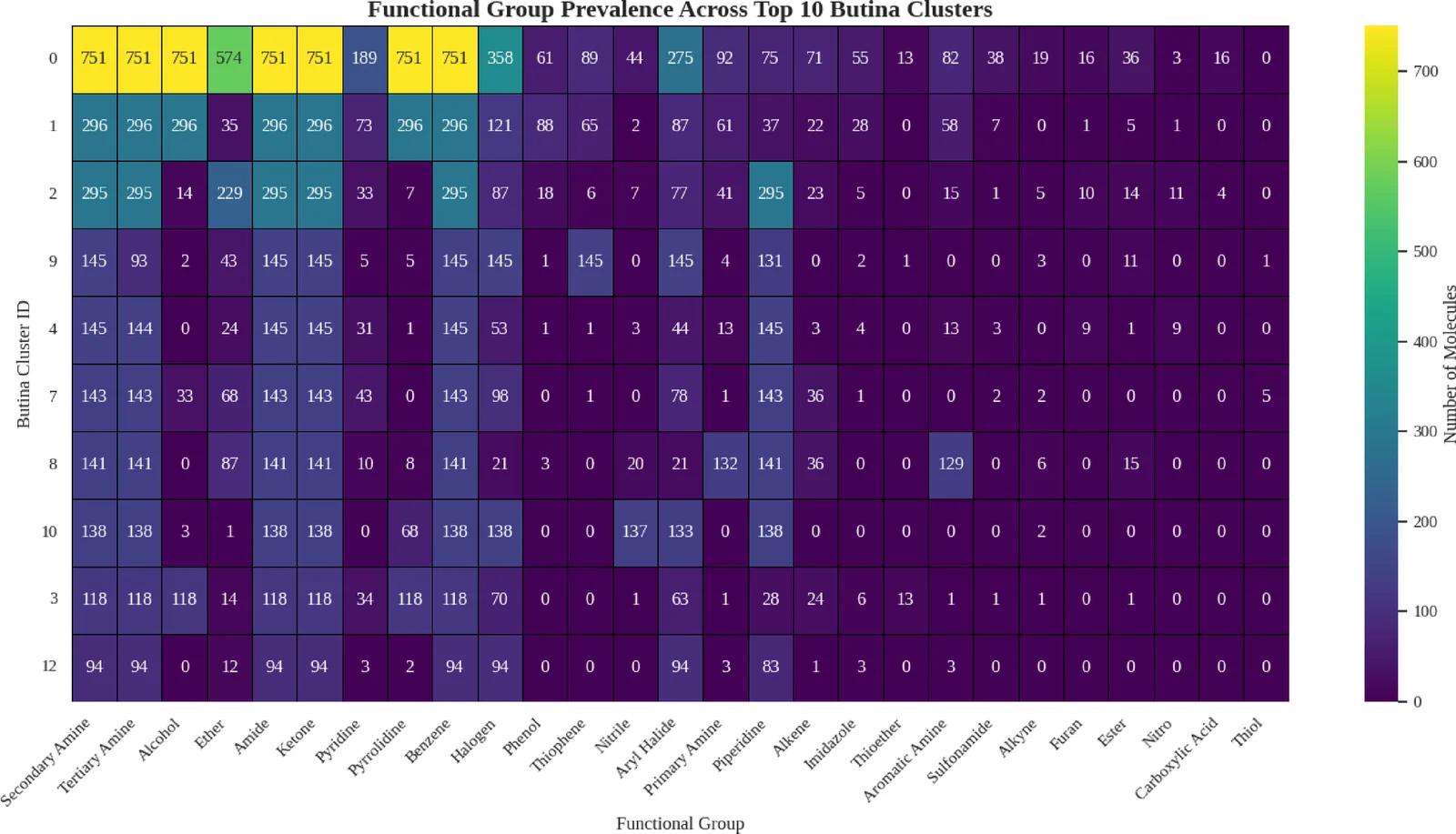
Heatmap showing the prevalence of functional groups across the top 10 Butina clusters, with colour intensity representing the number of molecules containing each functional group.

To gain deeper insight into the chemical features defining the PROTAC chemical space, a comprehensive functional group analysis was performed by screening each molecule for 27 common functional groups using SMARTS-based pattern matching. These functional groups, schematically illustrated to highlight their chemical identities (Fig. 12), represent key heteroatoms, heterocycles, and substituent classes frequently employed in medicinal chemistry and PROTAC design. The prevalence of each functional group within the top-10 frequent Butina clusters was quantified and visualized using a heatmap (Fig. 13), enabling direct comparison of functional group enrichment across chemically coherent clusters.

The heatmap in Fig. 13 reveals a pronounced enrichment of amine and amide functionalities across nearly all clusters. Secondary and tertiary amines, together with amide groups, are consistently observed at high frequencies, underscoring their central role in PROTAC molecular architectures. This trend is consistent with the chemical features observed in PROTACs currently in clinical trials. For example, ARV-110 and ARV-471 contain multiple amine- and amide-based linkages that facilitate attachment between the target-binding ligand, linker, and E3 ligase recruiter. Similarly, DT2216, a BCL-XL degrader, incorporates amide-rich linker motifs, while NX-2127, a BTK degrader, also relies on polar nitrogen-containing functionalities to achieve effective target engagement and degradation.

Functionally, amine and amide groups serve as key hydrogen-bond donors and acceptors, modulate molecular polarity, and provide chemically versatile points of attachment within PROTAC frameworks. Their widespread occurrence in frequent clusters aligns with the elevated hydrogen-bonding capacity observed in the physicochemical property analysis. It highlights the importance of polar interactions in stabilizing ternary complexes between target, PROTAC, and E3 ligase^15^. Collectively, these findings demonstrate that the functional group composition of the analyzed PROTAC chemical space closely mirrors the design principles underlying PROTACs that have advanced into clinical trials, thereby reinforcing the dataset’s translational relevance.

A substantial diversity of nitrogen‑containing heterocycles is also evident, including pyridine, imidazole, piperidine, and pyrrolidine motifs. As shown in Fig. 12, these heterocycles differ markedly in aromaticity, basicity, and three‑dimensional shape, allowing fine‑tuning of binding interactions and conformational preferences. The variable prevalence of these heterocycles across clusters in Fig. 13 suggests cluster‑specific scaffold preferences and target engagement strategies. For instance, clusters enriched in saturated heterocycles such as piperidine and pyrrolidine likely emphasize conformational flexibility and solubility, whereas aromatic heterocycles such as pyridine and imidazole may contribute to π‑stacking and directional hydrogen bonding^57,58^.

Oxygen-containing functional groups, including ethers, alcohols, phenols, esters, and carbonyl functionalities, are broadly distributed across the Butina clusters, reflecting the intrinsically polar and bifunctional architecture of PROTAC molecules. Among these, ether groups display substantial variability, ranging from less than 1% in Cluster 10 to approximately 60–78% in Clusters 0, 2, and 8 (e.g., ~ 62% in Cluster 8 and ~ 76% in Cluster 0). This marked enrichment is consistent with the frequent use of PEG-like linker motifs, which impart conformational flexibility and enable systematic tuning of linker length and spatial orientation between the warhead and the E3 ligase ligand. The high prevalence of ether functionalities enhances hydrogen-bond acceptor capacity and polarity while contributing to conformational plasticity, consistent with the elevated rotatable-bond counts observed in the same clusters.

Halogenated groups, encompassing both aliphatic and aryl halides, are also widely represented, with frequencies often exceeding 40–70% in several clusters (Fig. 13). Their consistent presence highlights their strategic role in medicinal chemistry for modulating lipophilicity, fine-tuning electronic distribution, reinforcing target binding via halogen bonding, and improving metabolic resilience. Beyond these broadly shared design elements, distinct cluster-specific signatures emerge. Cluster 3 is enriched in sulfur-containing functionalities such as thiols and thioethers, suggesting deliberate incorporation of sulfur atoms to increase polarizability, diversify interaction profiles, or introduce potential covalent or reversible covalent mechanisms. In contrast, Cluster 1 exhibits a pronounced thiophene signature, indicative of a sulfur-rich aromatic framework that distinguishes this series and may reflect unique linker architectures or target-binding scaffolds.

Taken together, the integration of qualitative visualization (Fig. 12) and quantitative distribution analysis (Fig. 13) offers a structured, data-driven depiction of functional group organization within PROTAC chemical space. The coexistence of widely distributed design motifs (ethers and halogens) alongside cluster-specific enrichments (sulfur-containing chemotypes) underscores both shared architectural principles and series-level differentiation. This stratification validates the effectiveness of similarity-based clustering in partitioning PROTACs into chemically coherent and mechanistically interpretable families, while simultaneously illuminating structure–property relationships relevant to molecular reactivity, conformational behaviour, binding interactions, and developability.

## Conclusion

This study addresses the challenge of understanding the chemical space of PROTAC-like compounds by systematically analyzing scaffold diversity, functional group composition, and key molecular features relevant to PROTAC design. Rdkit-based molecular representations combined with similarity-driven butina clustering (tanimoto cutoff 0.5) partitioned the curated dataset into 403 chemically meaningful clusters, with focused analysis of the top 10 most frequent groups. The clustering framework showed strong chemical coherence, supported by high intra-cluster Tanimoto similarity (0.748) and scaffold coherence (0.385), indicating that similarity-based clustering can robustly organize complex bifunctional molecules into interpretable structural families. structural analysis revealed diverse yet well-defined protac architectures, with recurrent murcko scaffolds capturing common E3 ligase-binding motifs, diverse target-binding warheads, and a broad range of linker chemotypes. Functional group profiling showed a consistently high prevalence of amine and amide functionalities across major clusters, while other groups, including sulfur-containing motifs, displayed pronounced cluster-specific patterns consistent with scaffold and design-dependent chemical choices. Physicochemical property analysis further indicated that protacs occupy a specialized chemical space that often exceeds conventional drug likeness ranges in molecular weight, lipophilicity, polarity, and flexibility, consistent with the requirements of ternary complex formation. Integration of E3 ligase recruiter and target protein annotations for the dominant clusters improved biological interpretability by linking chemically defined neighbourhoods to recruiter usage and recurrent target series. Overall, this work provides a data-driven organization of protac chemical space that supports informed scaffold selection and targeted molecular modification and offers a foundation for future integration with biological activity data and predictive modelling in targeted protein degradation research.

## Supplementary Information

Supplementary Information 1.

## Data Availability

The PROTAC dataset and all associated scripts and source codes used in this study are publicly available at: [ [https://github.com/ARTF-Lab-ML/PROTAC-Unsupervised-ML-Discovery](https:/github.com/ARTF-Lab-ML/PROTAC-Unsupervised-ML-Discovery) ]

## Abbreviations

PROTAC: Proteolysis-targeting chimera
ECFP: Extended-connectivity fingerprints
UMAP: Uniform manifold approximation and projection
HDBSCAN: Hierarchical density-based spatial clustering of applications with noise
PCA: Principal component analysis
SMILES: Simplified molecular input line entry system
RB: Rotatable bonds
