# Supplementary material for: Structural diversity and chemical space analysis of a PROTAC database using unsupervised machine learning
Source: Sci Rep. 2026 Jun 10;16:26372. doi: 10.1038/s41598-026-53215-x (PMC13500699; doi:10.1038/s41598-026-53215-x)
Supplement: Supplementary file 1 [file 41598_2026_53215_MOESM1_ESM.docx]

**Structural Diversity and Chemical Space Analysis of a PROTAC Database Using Unsupervised Machine Learning**

Ashutosh Kharwar ^a, b^, Alberto Marbán-González ^c^, José L. Medina-Franco ^c^, Carlos A. Velázquez-Martínez ^* a^

a	Faculty of Pharmacy and Pharmaceutical Science, University of Alberta, Edmonton, Alberta, T6G 2R3, Canada.

b	Department of Chemistry, Babasaheb Bhimrao Ambedkar University (A Central University), Lucknow, Uttar Pradesh, 226025, India.

c	DIFACQUIM Research Group, Department of Pharmacy, School of Chemistry, Universidad Nacional Autónoma de México, Mexico City, 04510, Mexico

Email: akharwar@ualberta.ca (Ashutosh Kharwar), velazque@ualberta.ca (Carlos A. Velázquez Martínez)

***Corresponding author**: Ashutosh Kharwar, Carlos A. Velázquez-Martínez

**Supplementary Information**

**Table S1.** Summary of clustering performance metrics (intra‑Tanimoto similarity, scaffold coherence, number of clusters, and silhouette score) for K‑means, hierarchical clustering, and HDBSCAN under different parameter settings.

| **Method** | **Intra-Tanimoto** | **Scaffold coherence** | **Number clusters** | **Silhouette Score** |
| --- | --- | --- | --- | --- |
| kmeans_k2 | 0.326 | 0.005 | 2 | 0.258 |
| kmeans_k3 | 0.317 | 0.006 | 3 | 0.158 |
| kmeans_k4 | 0.348 | 0.008 | 4 | 0.112 |
| kmeans_k5 | 0.38 | 0.019 | 5 | 0.122 |
| kmeans_k6 | 0.337 | 0.015 | 6 | 0.114 |
| kmeans_k7 | 0.409 | 0.023 | 7 | 0.121 |
| kmeans_k8 | 0.383 | 0.017 | 8 | 0.101 |
| kmeans_k9 | 0.367 | 0.018 | 9 | 0.111 |
| kmeans_k10 | 0.383 | 0.017 | 10 | 0.117 |
| Hierarchical_k2 | 0.323 | 0.005 | 2 | 0.239 |
| Hierarchical_k3 | 0.342 | 0.017 | 3 | 0.231 |
| Hierarchical_k4 | 0.439 | 0.024 | 4 | 0.213 |
| Hierarchical_k5 | 0.408 | 0.021 | 5 | 0.142 |
| Hierarchical_k6 | 0.415 | 0.021 | 6 | 0.095 |
| Hierarchical_k7 | 0.393 | 0.021 | 7 | 0.095 |
| Hierarchical_k8 | 0.388 | 0.023 | 8 | 0.098 |
| Hierarchical_k9 | 0.388 | 0.022 | 9 | 0.103 |
| Hierarchical_k10 | 0.407 | 0.022 | 10 | 0.108 |
| HDBSCAN | 0.587 | 0.03 | 3 | 0.146 |
| HDBSCAN_min10 | 0.803 | 0.088 | 148 | 0.463 |
| HDBSCAN_min20 | 0.739 | 0.069 | 73 | 0.412 |
| HDBSCAN_min30 | 0.587 | 0.03 | 3 | 0.146 |
| HDBSCAN_min50 | 0.448 | 0.011 | 2 | 0.2 |
| HDBSCAN_min100 | 0.371 | 0.022 | 4 | 0.237 |
| Butina_cutoff0.4 | 0.824 | 0.445 | 670 | - |
| Butina_cutoff0.5 | 0.748 | 0.385 | 403 | - |
| Butina_cutoff0.6 | 0.645 | 0.337 | 174 | - |
| Butina_cutoff0.7 | 0.53 | 0.285 | 61 | - |

**Figure S1.** E3 ligase recruiter distribution across the top 10 Butina clusters from (a) to (j).

**Figure S2.** Target distribution across the top 10 Butina clusters from (a) to (j).
